# Protein expression changes of HCN1 and HCN2 in hippocampal subregions of gerbils during the normal aging process

**DOI:** 10.22038/ijbms.2019.35760.8520

**Published:** 2019-11

**Authors:** Choong-Hyun Lee, Joon Ha Park, Moo-Ho Won

**Affiliations:** 1 Department of Pharmacy, College of Pharmacy, Dankook University, Cheonan, Chungnam 31116, Republic of Korea; 2 Department of Anatomy, College of Korean Medicine, Dongguk University, Gyeongju, Gyeongbuk 38066, Republic of Korea; 3 Department of Neurobiology, School of Medicine, Kangwon National University, Chuncheon, Gangwon 24341, Republic of Korea

**Keywords:** Aging, Dentate gyrus, Granule cells, HCN channel, Hippocampus proper, Pyramidal cells

## Abstract

**Objective(s):**

Hyperpolarization-activated cyclic nucleotide-gated (HCN) channels play essential roles in various hippocampal functions, including regulation of long-term potentiation, synaptic plasticity, and hippocampal-dependent cognitive process. The objective of this study was to investigate age-related changes in HCN1 and HCN2 protein expressions in gerbil hippocampus at various ages.

**Materials and Methods:**

In this study, the protein expressions of HCN1 and HCN2 were compared in the hippocampus at the ages of 1, 3, 12, and 24 months using Western blot analysis and immunohistochemistry.

**Results:**

Immunoreactivity of both HCN1 and HCN2 was shown primarily in cells of the pyramidal cell layer in the hippocampus proper and in cells of the granule cell layer in the dentate gyrus. HCN1 and HCN2 protein expression levels and immunoreactivity were significantly increased at three months (3 M) of age compared with those at 1 M of age. After that, both HCN1 and HCN2 expression levels in the hippocampus were gradually decreased with age.

**Conclusion:**

Our results show that the normal aging process affects the expression levels of HCN1 and HCN2 in hippocampal cells in gerbils. There are marked reductions in HCN1 and HCN2 expressions in the aged hippocampus compared to the young hippocampus. Such reductions might be related to aging in the hippocampus.

## Introduction

Normal aging-associated cognitive decline, memory loss, and abnormal alterations in the brain have been thought to be the most important feature in the elderly population (1). The hippocampus in the brain plays an essential role in processes for learning and memory. It is known as one of the most vulnerable brain regions during the normal aging process (2, 3). It is also well known that aging-associated cognitive decline is related to structural and functional changes in the hippocampus such as loss of neurons, alteration of synaptic plasticity, and impairment in the induction of long-term potentiation (4-6).

Hyperpolarization-activated cyclic nucleotide-gated (HCN) channels that conduct hyperpolarization-activated cation current (Ih) are involved in various neural functions such as the regulation of resting membrane potential, neural rhythmic activity, long-term potentiation, and synaptic transmission (7-10). In addition, HCN channels are critical modulators of hippocampal-based learning and memory (11-13). 

Until now, four subunits (HCN1-4) of a gene family encoding HCN channels have been identified (14, 15). Among them, HCN1 is abundantly expressed in pyramidal neurons of the neocortex and hippocampus, and it responds minimally to cyclic adenosine monophosphate (cAMP) (16, 17). HCN2 is widely expressed throughout the brain and conducts Ih currents with slower kinetics compared to HCN1. However, it responds strongly to cAMP (14, 17, 18). Recently, it has been reported that HCN1 and HCN2 are strongly and substantially expressed in the hippocampi of rats and mice (19-22). Besides, HCN channels are differentially expressed in the hippocampus during postnatal development of rats and mice (19, 20, 22-24). However, age-related changes of expressions of HCN channels in aged brains have not been fully elucidated yet. Therefore, the objective of the present study was to investigate age-dependent changes in HCN1 and HCN2 expression in gerbil hippocampus during the normal aging process using Western blot and immunohistochemistry analyses.

## Materials and Methods

***Experimental animals***

This study used male Mongolian gerbils (Meriones unguiculatus) at the ages of 1 (25–30 g), 3 (40–50 g), 12 (65–75 g), and 24 (85–95 g) months (1 M, 3 M, 12 M, and 24 M) according to their suitability for aging research (20). The gerbils (n=14 animals in each group) were obtained from the Experimental Animal Center of Kangwon National University, Chuncheon, Gangwon, Republic of Korea. They were housed under conventional conditions at ambient temperature (23ie ^°^C) and relative humidity (55±5%) under a 12-hr light/dark cycle and were allowed free access to food and water. The procedures for animal handling and care adhered to guidelines in compliance with the current international laws and policies (Guide for the Care and Use of Laboratory Animals, The National Academies Press, 8th ed., 2011). Procedures of all experiments, including animal handling and use, were reviewed and approved (approval no. KW-160802-2) by Institutional Animal Care and Use Committee (IACUC) of Kangwon National University.

***Western blot analysis***

hanges in HCN1 and HCN2 protein levels were examined in hippocampi obtained from each group (n= 7 animals in each group) at the age of 1 (25–30 g), 3 (40–50 g), 12 (65–75 g), and 24 (85–95 g) M by Western blot analysis according to our published papers (25, 26). In brief, the gerbils were anesthetized with zoletil 50 (60 mg/kg; Virbac. Carros, France) (27), sacrificed by cervical dislocation and decapitated. Their brains were removed and cut serially and transversely into 400 µm thick sections using a vibratome (Leica Camera AG, Wetzlar, Germany). Subsequently, the hippocampi were dissected out from the brain sections by using a surgical blade and fine forceps. The hippocampal tissues were homogenized in 50 mM phosphate-buffered saline (PBS; pH 7.4) containing 0.1 mM ethylene glycol-bis(2aminoethyl ether)N,N,N’,N’tetraacetic acid (pH 8.0), 0.2% Nonidet P-40, 10 mM ethylenediaminetetraacetic acid (pH 8.0), 15 mM sodium pyrophosphate, 100 mM β-glycerophosphate, 50 mM NaF, 150 mM NaCl, 2 mM sodium orthovanadate, 1 mM phenylmethylsulfonyl ﬂﬂﬂid , and 1 mM dithiothreitol (DTT; all from Sigma-Aldrich; Merck KGaA, Darmstadt, Germany). Following centrifugation at 16,000 x g for 20 min at 4 ^°^C, the HCN1 and HCN2 protein expression levels in the supernatants were determined using a micro bicinchoninic acid protein assay kit (Sigma-Aldrich; Merck KGaA). Aliquots containing 20 µg total protein were loaded onto a 10% polyacrylamide gel. Following electrophoresis, the gels were transferred onto nitrocellulose transfer membranes. The membranes were incubated with rabbit anti-HCN1 (AB5884; 1:200, Millipore, Temecula, CA) or rabbit anti-HCN2 (AB5378; 1:200, Millipore) or mouse anti-β-actin (A5316; 1:5,000, Sigma-Aldrich; Merck KGaA) overnight at 4 ^°^C. Moreover, the membranes were incubated with peroxidase-conjugated donkey anti-rabbit immunoglobulin G (IgG; sc-2305; 1:1,000; Santa Cruz Biotechnology, Inc., Dallas, TX, USA) for one hr at room temperature, followed by ECL reagents (Pierce; Thermo Fisher Scientifc, Inc.). The resulting protein bands were scanned, and densitometric analysis for quantification of the bands was performed using Image J 1.59 software (National Institutes of Health, Bethesda, MD, USA), which was used to calculate the relative optical density (ROD). The protein levels of HCN1 and HCN2 were normalized to that of β-actin. A ratio of the ROD was calibrated as a percentage, with the 1 M group designated as 100%.

***Immunohistochemistry***

or tissue preparation, the gerbils (n=7 animals in each group) were anesthetized with zoletil 50 (60 mg/kg; Virbac) (27) and fixed by perfusion via the ascending aorta with 4% paraformaldehyde in 0.1 M phosphate-buffered saline (PBS, pH 7.4). Their brains were removed and postfixed with the same fixative for 12 hr. Brain tissues, including hippocampi, were sectioned into 30-μm thickness in a cryostat.

To examine age-related changes of HCN1 and HCN2 immunoreactivity in the hippocampus, immunohistochemical staining was performed according to the method described in our published papers (25, 26). In brief, the sections were incubated with rabbit anti-HCN1 (1:100, Millipore) or rabbit anti-HCN2 (1:100, Millipore) as primary antibodies overnight at 4 ^°^C. Following three washes with PBS (each for 10 min), the reacted tissues were exposed to biotinylated goat anti-rabbit IgG (BA-1000; 1:200; Vector Laboratories, Burlingame, CA, USA) for 2 hr at room temperature and then streptavidin peroxidase complex (SA-5004; 1:200; Vector Laboratories) for 45 min at room temperature. Finally, the reacted sections were visualized with 3,3’-diaminobenzidine. To establish the specificity of the immunostaining, a negative control test was performed using a pre-blocking serum (S-1000; Vector Laboratories, Inc.) instead of primary antibody, which resulted in the absence of immunoreactivity in all structures (data not shown).

For the quantitative analysis of HCN1 and HCN2 immunoreactivity, six sections per animal were selected with a 120-μm interval. Digital images of HCN1 and HCN2 immunoreactivity were captured with an Axio Imager 2 microscope (Carl Zeiss, Germany) equipped with a digital camera (Axiocam, Carl Zeiss) connected to a PC monitor. According to our published method (25, 26, 28), the semi-quantification HCN1 and HCN2 immunoreactivity in cells of the pyramidal cell layer in the hippocampus proper and in cells of the granule cell layer in the dentate gyrus were evaluated as relative immunoreactivity (RI) by using NIH Image J 1.59 software. The images were calibrated into an array of 512 x 512 pixels corresponding to a tissue area of 250 x 250 µm (x20 primary magnification). The mean immunoreactivity of HCN1 and HCN2 in the immunoreactive structures was measured by a 0–255 grayscale system (white to dark signal corresponded from 255 to 0). After the background was subtracted, and the staining intensity was calculated. A ratio of the RI in the immunoreactive structures was also represented: a ratio of the RI was calibrated as %, with the 1 M group designated as 100%.

**Figure 1:**
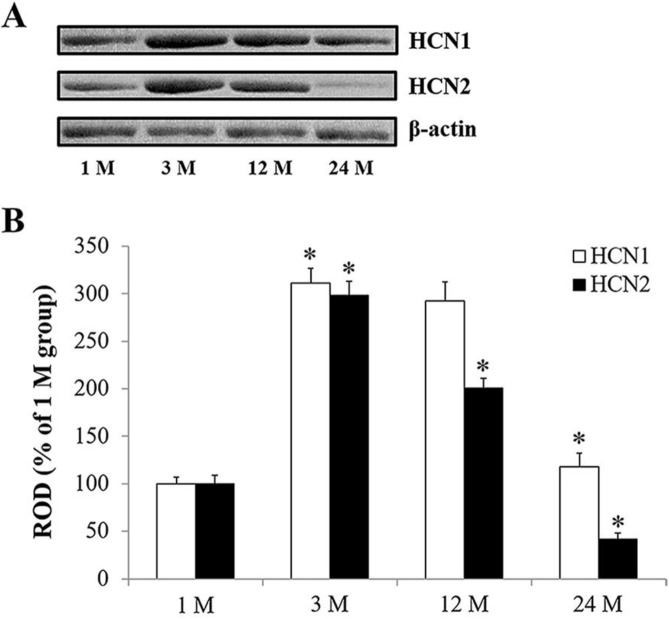
(A) Western blot analyses of HCN1 and HCN2 in the hippocampus at various ages. (B) The ROD of immunoblot band is demonstrated as % values (n=7 per group; *P<0.05, significantly different from each pre-time point group). Data are presented as means±SEM

**Figure 2:**
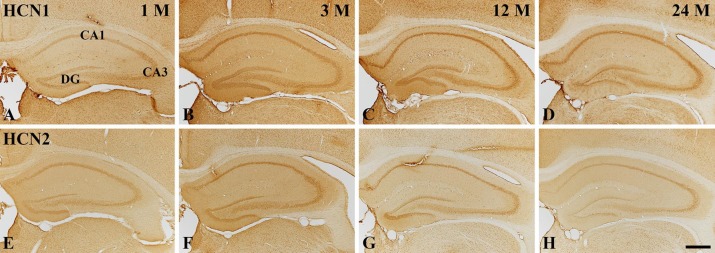
Low magnification of immunohistochemistry for HCN1 (A-D) and HCN2 (E-H) in the gerbil hippocampus at 1 M (A, E), 3 M (B, F), 12 M (C, G), and 24 M (D, H). CA, cornu ammonis; DG, dentate gyrus. Scale bar = 800 μm

**Figure 3:**
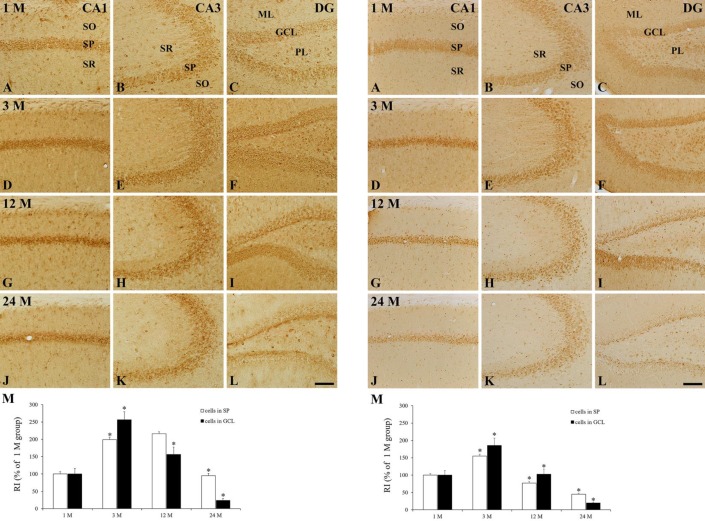
HCN1 immunohistochemistry in the CA1 (A, D, G, J) and CA3 (B, E, H, K) regions, and dentate gyrus (C, F, I, L) of the 1 M (A-C), 3 M (D-F), 12 M (G-I), and 24 M (J-L) group. HCN1 immunoreactivity is shown primarily in cells of the striatum pyramidale (SP) of the CA1–3 regions and in the granule cell layer (GCL) of the dentate gyrus. HCN1 immunoreactivity in the SP and GCL is significantly increased at 3 M and gradually decreased after that. ML, molecular layer; PL, polymorphic layer; SO, stratum oriens; SR, stratum radiatum. Scale bar = 100 μm. M: RI as % of HCN1 immunoreactivity in cells in the SP and GCL (n = 7 per group; *P<0.05, significantly different from the pre-time point group). Data are presented as means±SEM

**Figure 4:**
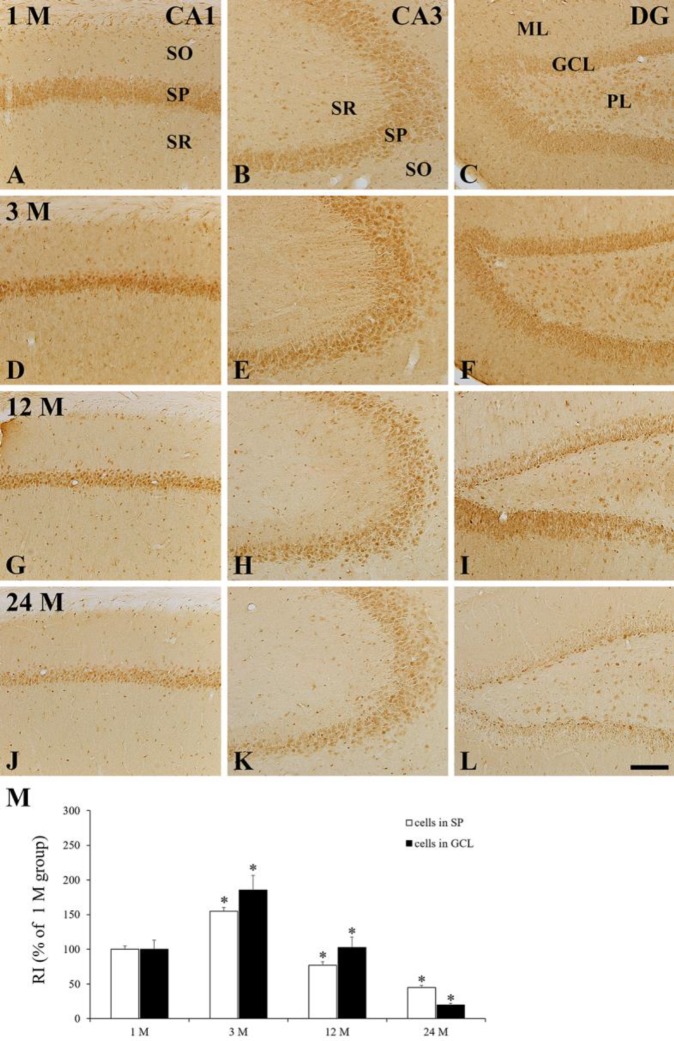
Immunohistochemistry of HCN2 in the CA1 (A, D, G, J) and CA3 (B, E, H, K) regions, and dentate gyrus (C, F, I, L) at 1 M (A-C), 3 M (D-F), 12 M (G-I), and 24 M (J-L). HCN2 immunoreactivity is found primarily in cells of the SP of the CA1-3 regions and in the GCL of the dentate gyrus. At 3 M, HCN2 immunoreactivity is significantly increased in the SP and GCL and gradually decreased thereafter. ML, molecular layer; PL, polymorphic layer; SO, stratum oriens; SR, stratum radiatum. Scale bar=100 μm. M: RI as % of HCN2 immunoreactivity in cells in the SP and GCL (n=7 per group; *P<0.05, significantly different from the pre-time point group). Data are presented as means ± SEM

***Statistical analysis***

Data are expressed as the mean ± SEM. The difference of the mean RI among the groups was statistically analyzed using a one-way ANOVA followed by a *post hoc* Bonferroni’s multiple comparison tests. Statistical significance was considered at *P*<0.05.

## Results

***Changes in HCN1 and two protein levels***

rom the Western blot analysis (Figures 1A and 1B), we found that both HCN1 and HCN2 protein levels in gerbil hippocampus were significantly increased in the 3 M group compared to the 1M group (270.0 % and 208.7 % of the 1 M group, respectively). Then, HCN1 protein level was maintained until 12 M and significantly decreased at 24 M compared with that at 12 M (40.3 % of the 12 M group). On the other hand, HCN2 protein level at 12 M was significantly decreased compared with that at 3 M (67.2 % of the 3 M group). HCN2 protein at 24 M was more significantly decreased and significantly lower than that at 12 M (41.6 % of the 1 M group).

***Change in HCN1 immunoreactivity***

Hippocampus proper (CA1-3 regions): in the 1 M group, moderate HCN1 immunoreactivity was readily detected in pyramidal cells of the stratum pyramidale (SP) in the hippocampus proper, and HCN1 immunoreactivity was shown in non-pyramidal cells of the stratum oriens (SO) and stratum radiatum (SR) (Figures 2A, 3A, and 3B). In the 3 M group, HCN1 immunoreactivity in cells of the SP was significantly increased by 199.1 %, compared to that in the 1M group (Figures 2B, 3D, 3E, and 3M). HCN1 immunoreactivity in the SP at 12 M was similar to that at 3 M (Figures 2C, 3G, 3H, and 3M). At 24 M, HCN1 immunoreactivity in the SP was significantly decreased by 44.0 %, compared to the 12 M group (Figures 2D, 3J, 3K, and 3M).

Dentate gyrus: in the 1 M group, HCN1 immunoreactivity in the dentate gyrus was detected primarily in granule cells of the granule cell layer (GCL), and in some polymorphic cells of the polymorphic layer (PL) (Figures 2A and 3C). At 3 M, HCN1 immunoreactivity in the GCL was dramatically increased: the RI was 256.4 % of the 1 M group (Figures 2B, 3F, and 3M). In the 12 M group, HCN1 immunoreactivity in the GCL was significantly decreased by 61.1 %, compared with that of the PM 3 group (Figures 2C, 3I, and 3M). At 24 M, HCN1 immunoreactivity in the GCL was more significantly decreased (Figures 2D, 3L, and 3M).

***Change in HCN2 immunoreactivity***

Hippocampus proper (CA1–3 regions): in the 1 M group, HCN2 immunoreactivity was detected primarily in cells of the SP, and some cells in the SO and SR showed HCN2 immunoreactivity (Figures 2E, 4A, 4B, and 4M). At 3 M, like the change of HCN1 immunoreactivity in the hippocampus proper, HCN2 immunoreactivity in the SP was significantly increased by 155.1 % compared to that in the 1 M group (Figures 2F, 4D, 4E, and 4M). At 12 M, unlike the change of HCN1 immunoreactivity in the hippocampus proper, HCN2 immunoreactivity of the SP was decreased by 49.8 % compared to that in the 3 M group (Figures 2G, 4G, 4H, and 4M). In the 24 M group, HCN2 immunoreactivity was more decreased: the RI was 58.3 % of the 12 M group (Figures 2H, 4J, 4K, and 4M).

entate gyrus: Change pattern of HCN2 immunoreactivity in the dentate gyrus was similar to the change of HCN1 immunoreactivity (Figure 4M). At 3 M, the ROD of HCN2 immunoreactivity in the GCL was 185.3 % of that at 1 M (Figures 2B, 4F, and 4M). After that, HCN2 immunoreactivity was gradually decreased with time, and the RI of the 24 M group was 19.2 % of that of the 12 M group (Figures 2G, 2H, 4I, 4L, and 4M). 

## Discussion

ome previous studies have demonstrated the developmental regulation of HCN channels expressions in the rodent hippocampus. Vasilyev and Barish (24) have reported that both HCN1 and HCN2 immunoreactivities are significantly increased in pyramidal neurons in CA1 and CA3 regions of the mouse hippocampus up to postnatal day 20 (P20) compared to those at P1 and P5. They have suggested that changes in numbers and structures of HCN channels might be associated with developmental increases in Ih amplitude and activation rate (24). In addition, Surges *et al*. (23) have shown that mRNA and protein levels of HCN1 and HCN2 are significantly increased in pyramidal neurons of the rat hippocampal CA1 region during the first four weeks after birth. In the present study, we found that protein levels and immunoreactivities of HCN1 and HCN2 were dramatically increased in cells of the stratum pyramidale (pyramidal cells or neurons) in CA1–3 regions and in cells of the granular layer (granule cells) in the dentate gyrus at 3 M compared to those at 1 M. This result is in line with the result of a previous study that shows that HCN1 protein levels are increased in the rat hippocampus between P11 and P90 and that HCN2 protein levels are increased robustly during the first two weeks after birth (20). Therefore, based on our present result with the previous studies, expressions of HCN1 and HCN2 protein might likely increase in the hippocampus in early phase during the normal aging process.

In this study, we observed that both HCN1 and HCN2 protein expressions in the hippocampus were markedly decreased in the 24 M group compared to those in the 3 M and 12 M groups. HCN channels have been thought to be closely involved in constraining long-term potentiation and contributing to synaptic integration, plasticity, and transmission in the hippocampus (8, 29, 30). Furthermore, it has been reported that HCN channels, especially the HCN1 channel, might play essential roles in modulating learning and memory (12). Also, it has been widely accepted that synaptic plasticity and long-term potentiation in the hippocampus and cognitive function are decreased age-dependently during the normal aging process (31-35). Therefore, an age-related decrease of HCN1 and HCN2 protein expression in the aged hippocampus might be related to age-dependent cognitive impairment, alterations of long-term potentiation, and synaptic transmission.

Another notable finding in this study is that there were differences in age-related decreases in HCN1 and HCN2 protein expressions in the hippocampus. Namely, HCN1 immunoreactivity was significantly decreased in the dentate gyrus, not in the hippocampus proper at 12 M, and HCN2 immunoreactivity was significantly decreased in both hippocampus proper and dentate gyrus at 12 M compared with that at 3 M. This is the first finding that suggests that HCN1 and HCN2 expressions are differently affected according to hippocampal subregions in the normal aging process of the hippocampus. It has been demonstrated that hippocampal subregions are differently affected by the normal aging process. The dentate gyrus is the most vulnerable subregion in the hippocampus during the aging process; whereas the hippocampus proper is relatively resistant to the aging process (36). It has also been suggested that age-related cognitive impairment begins to occur at around 12 M in mice (35, 37). In addition, some previous studies have reported that imbalance between HCN1 and HCN2 expression can lead to impaired learning and memory (38, 39). Therefore, it can be postulated that the differential decrease of HCN1 and HCN2 immunoreactivity between the dentate gyrus and the hippocampus proper from 12 M might be associated with the start of age-related cognitive decline and age-dependent vulnerability-related subregional heterogeneity in the hippocampus.

## Conclusion

Our present study showed that both HCN1 and HCN2 protein expression levels were differently altered according to hippocampal subregions during the normal aging process and significantly decreased in aged hippocampus, although the limitations of this study include the lack of data on age-related changes in HCN1 and HCN2 gene expressions as well as definite relationship between reduced HCN expression and altered hippocampal functions in aged animals, which should be addressed in future studies. The findings indicate that significant reductions of HCN1 and HCN2 protein expressions in the aged gerbil hippocampus might be related to the normal aging process in the hippocampus.
